# Anandamide Alters Barrier Integrity of Bovine Vascular Endothelial Cells during Endotoxin Challenge

**DOI:** 10.3390/antiox11081461

**Published:** 2022-07-27

**Authors:** Carsten C. F. Walker, Lorraine M. Sordillo, G. Andres Contreras

**Affiliations:** Department of Large Animal Clinical Sciences, College of Veterinary Medicine, Michigan State University, East Lansing, MI 48824, USA; sordillo@msu.edu (L.M.S.); contre28@msu.edu (G.A.C.)

**Keywords:** endothelial cells, endocannabinoids, arachidonoylethanolamide, anandamide, cannabinoid receptor-1, lipopolysaccharide, barrier integrity, inflammation, reactive oxygen species, oxidative stress

## Abstract

Vascular endothelial cells are crucial mediators of inflammation during infectious diseases, due to their ability to produce lipid-based inflammatory mediators and facilitate leukocyte migration and translocation to infected tissues. Mastitis is the costliest infectious disease in North America, with over two billion dollars in annual costs due to loss of milk production, medical treatment, and potential loss of the animal. Infections caused by coliform bacteria are particularly deleterious, causing a negative impact on cow well-being and a high mortality rate. Dysfunction and breakdown of the endothelial barrier is a key part of the pathology of coliform mastitis. The endocannabinoid system (ECS), shown to modulate inflammatory responses of vascular endothelial cells in humans and rodents, may be a novel target for inflammatory modulation in dairy cows. The endocannabinoid (EC) arachidonoylethanolamide (AEA) is a potent anti- or pro-inflammatory mediator in endothelial cells, depending on location, timing, and concentration. We hypothesized that elevated AEA during LPS challenge will impair endothelial barrier integrity via increased production of reactive oxygen species (ROS) and activation of apoptotic pathways. Challenge of bovine aortic endothelial cells (BAEC) with 25 ng/mL lipopolysaccharide (LPS) for 8 h induced AEA synthesis, increased expression of cannabinoid receptor 1 and 2 (CB1/2) and the AEA synthesizing enzyme N-acyl phosphatidylethanolamine phospholipase D (NAPE-PLD), while decreasing gene expression of the AEA degradation enzyme fatty acid amide hydrolase (FAAH). Trans endothelial resistance (TER), measured through electrical resistance across the monolayer, increased 2 h after 0.5 µM AEA treatment and decreased with 5 µM AEA, compared to LPS alone. Addition of AEA to BAEC challenged with LPS induced mitochondrial dysfunction via increased ROS production, cytochrome-C release, and activation of caspase 3/7. Antagonism of CB1 by 1 µM AM251 ameliorated AEA induced ROS production and cytochrome-C release. Addition of AM251 also eliminated 2 h TER increase and improved TER following 5 µM AEA. Doses of 0.5, 1, and 5 µM AEA delayed endothelial barrier recovery, which was eliminated by the addition of AM251. Mitochondrial dysfunction and activation of apoptotic pathways in response to AEA treatment during LPS challenge of BAEC may act to delay inflammatory resolution and contribute to endothelial dysfunction.

## 1. Introduction

Infection of the mammary gland, or mastitis, is one of the diseases with the greatest impact on animal welfare and dairy cow health in North America [1,2]. Infections caused by coliform bacteria can be particularly severe, with a negative impact on cow well-being and a mortality rate of over 30% [3,4,5]. Effective clearance of the pathogen is highly dependent on a properly regulated inflammatory response, with rapid onset and timely resolution, to prevent excessive mammary secretory tissue damage. However, acute infections are associated with overproduction of pro-inflammatory oxylipids, excessive leukocyte infiltration into infected tissue, and excessive reactive oxygen species’ (ROS) production that may lead to oxidative stress, damaging healthy tissues.

Breakdown of the epi- and endothelial monolayers is part of the pathology of coliform mastitis in dairy cows [6]. Stimulation of the Toll-like receptor 4 (TLR4) by the coliform derived bacterial endotoxin lipopolysaccharide (LPS), results in increased expression of adhesion molecules, as well as increased production of inflammatory mediators, such as oxylipids, cytokines, and ROS. Inflammatory responses during coliform mastitis include synthesis of pro- and anti-inflammatory oxylipids that were recently characterized in in vitro models of endothelial inflammation. Increased synthesis of specific oxylipids contributes to endothelial cell dysfunction and reduction in barrier integrity [7], whereas others improve integrity and act to resolve inflammation [8].

Closely tied to oxylipids, are a class of potent lipid-based inflammatory mediators called endocannabinoids (EC). As part of the endocannabinoid system (ECS), EC and dedicated cannabinoid receptors-1 and -2 (CB1/2), are involved in several physiological processes, such as energy homeostasis, cellular metabolism, pain, and inflammation [9]. Fluctuations of EC plasma concentrations during inflammatory events in dairy cows has recently been demonstrated [10] and elevated concentrations of arachidonoylethanolamide, or anandamide (AEA) associated with non-steroidal anti-inflammatory drug (NSAID) treatment in murine models, is theorized to contribute to the analgesic effects of the drugs [11]. Considering that in isolated endothelial and other immune mediating cells, effects of AEA not only vary by tissue type but are highly time and concentration dependent [12], increased AEA concentrations can lead to elevated ROS production, mitochondrial dysfunction, prolonged activation of macrophages and neutrophils, as well as induction of apoptotic signaling via cannabinoid receptors 1 and 2 (CB1/2) [13]. Therefore, untimely elevation of AEA may contribute to the dysfunctional inflammatory response through prolonged pro-inflammatory signaling in dairy cows. As administration of NSAIDs in dairy cattle suffering from acute coliform mastitis occurs only when symptoms have become systemic, the increase in AEA concentration associated with NSAID treatment may result in further deterioration of the endothelial barrier. However, studies pertaining to the effects of the ECS on endothelial barrier function have focused on pre-treatment with EC and concurrent LPS challenge and EC administration, which does not consider the possibility of significant changes in ECS receptor and enzyme expression that are well documented in immune mediating cells [13].

Therefore, we set out to characterize the effects of AEA on BAEC function and barrier integrity, during LPS challenge. We demonstrate that the ECS is not only present in BAEC, but that expression of key genes is altered by LPS challenge. We also demonstrate that CB1 activation by AEA concentrations as low as 0.5 µM, are detrimental to BAEC function and monolayer barrier integrity, during LPS challenge. Lastly, we demonstrate that AEA induces ROS production and the likelihood of oxidative stress in BAEC without LPS challenge, while apparent mitochondrial dysfunction results from AEA treatment of BAEC during LPS challenge, leading to activation of apoptotic pathways.

## 2. Materials and Methods

### 2.1. Reagents

High performance liquid chromatography (HPLC) grade acetonitrile, HPLC-grade methanol, formic acid, glycerol, transferrin, insulin, heparin, sodium selenite, Hanks buffered salt solution (HBSS) powder, collagenase, lipopolysaccharide (LPS) (O111:B4), 156 EDTA, and tri-phenylphosphine were purchased from Sigma-Aldrich (Burlington, MA, USA). Deuterated and nondeuterated isoprostane standards, indomethacin, 2,2′-azobis-2-methyl-propanimidamide dihydrochloride (AAPH), arachidonoylethanolamide (AEA) and AM251 were purchased from Cayman Chemical (Ann Arbor, MI, USA). Butylated hydroxy toluene (BHT) was purchased from Acros (Waltham, MA, USA). Fetal bovine serum was purchased from Hyclone Laboratories, Inc. (Logan, UT, USA) and 4-(2-hydroxyethyl)-1-piperazineethanesulfonic acid (HEPES) buffer, and dimethyl sulfoxide (DMSO) were purchased from Corning Inc. (Corning, NY, USA). HAM’s F-12k was purchased from Irvine Scientific (St. Santa Ana, CA, USA). Antibiotics/antimycotics, trypsin-EDTA, bovine collagen, and ProLong-Gold antifade were from Life Technologies (Carlsbad, CA, USA). Predesigned and custom-made bovine TaqMan primers were purchased from Applied Biosystems (Foster City, CA, USA). 4′,6-diamidino-2-phenylindole (DAPI) was purchased from Molecular Probes (Eugene, OR, USA), and von Willebrand’s factor was purchased from Agilent Technologies (Santa Clara, CA, USA).

### 2.2. Primary Cell Isolation

Endothelial barrier function was assessed using primary bovine aortic endothelial cells collected as described previously [14]. Briefly, for BAEC isolation, a 10 cm full-circumference section of descending aorta, just distal to the branch of the subclavian artery, was collected at a commercial abattoir and immediately submerged in HBSS with 0.05 mg/mL gentamicin on ice. Once transported to the laboratory for processing, aorta samples were cut lengthwise and laid flat in a solution of 2 mg/mL collagenase in Krebs-Ringer bicarbonate with 4% BSA and allowed to incubate at 37 °C for 10 min. After each incubation, the collagenase solution was collected and the luminal sides of tissues were rinsed with HBSS, collecting the rinse solution. The rinse solution was added to collected collagenase and samples were centrifuged at 160× *g* for 10 min at 15 °C. Resulting cellular pellets were plated in T25 cell culture flasks until confluent in Ham’s F12K medium containing 10% fetal bovine serum, 10 mM HEPES buffer, 0.25% sodium bicarbonate, 2 mM L-glutamine, 1% 1:1 antibiotic:antimycotic, 100 μg/mL heparin, 10 μg/mL insulin, 5 μg/mL transferrin, and 40 ng/mL sodium selenite. Cells were detached via incubation with 0.05% trypsin, diluted serially, and plated in 96-well plates. To exclude fibroblasts and other cell types, wells containing only colonies derived from a single cell were selected for propagation. Wells were selected by typical endothelial cobblestone morphology for further propagation and confirmed by von Willebrand factor staining. Cells were frozen at passage 3, grown to passage 6 for RNA isolation, representing the earliest passage at which cells could realistically be used for assays, and discarded after passage 9.

### 2.3. Experimental Design

**Figure 1:**
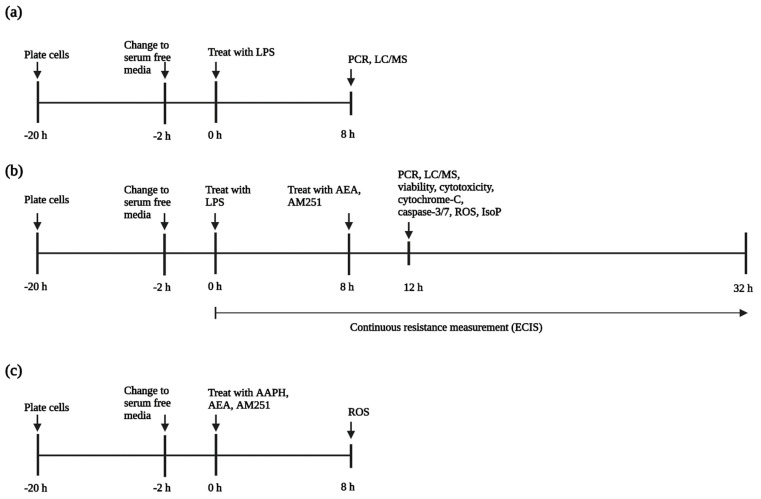
Experimental design to determine the effect of arachidonoylethanolamide (AEA) on bovine aortic endothelial cells (BAEC) (N = 6). (**a**) Initial experiment to establish presence of genes related to the endocannabinoid system and concentrations of AEA in response to lipopolysaccharide (LPS) challenge; (**b**) Experimental design to establish effect of AEA on gene expression, isoprostane production, viability, cytotoxicity, cytochrome-C release, and caspase 3/7 activation in BAEC; (**c**) Experimental design to establish effect of AEA on reactive oxygen species’ (ROS) production without LPS challenge. Figure created with Biorender.com. AAPH: 2,2′-azobis (2-amidinopropane) dihydrochloride; AM251: 1-(2,4-dichlorophenyl)-5-(4-iodophenyl)-4-methyl-N-1-piperidinyl-1H-pyrazole-3-carboxamide; LC/MS: liquid chromatography/mass-spectrometry; PCR: polymerase chain reaction.

Cells were plated at 1 × 10^5^ cells/well in 96 well plates, and 1 × 10^6^ cell/well in 6 well plates, and grown to confluency in 5% FBS Ham’s F12k media containing 20 mM HEPES, antibiotics and antimycotics (100 U/mL consisting of penicillin, streptomycin, and amphotericin B), heparin (100 µg/mL), insulin (10 µg/mL), transferrin (5 µg/mL), and sodium selenite (10 ng/mL), for 18 h before media was changed to serum free media for 2 h (Figure 1). Treatments were as follows: untreated media control, vehicle control with 0.05% ethanol by volume, positive control of either 25 ng/mL LPS or 3 mM AAPH as an agonist, physiologically relevant doses of AEA (0.5, 1, and 5 µM), 1 µM of the CB1 antagonist AM251, and co-culture of either agonist and AEA, with and without AM251. To mimic coliform infections, cells were pre-treated with 25 ng/mL LPS + vehicle for 8 h before addition of AEA and AM251. Results were recorded at 2, 4, 8, 12, and 24 h post-AEA addition, with the 4 h time point showing the greatest change in results. Only the 4 h time point is presented here; the remaining time points can be found in the Supplemental Data. All experiments were carried out with an N = 6.

### 2.4. Real-Time qPCR

Collection of cells was initiated by washing twice with HBSS and 300 μL of buffer RLT (Qiagen, Germantown, MD, USA) was added for cell lysis. Buffer RLT was collected and stored at −20 °C for no longer than one month before processing. Extraction of RNA occurred utilizing a Promega Maxwell RSC Instrument following the manufacturer’s protocol. RNA quantity and quality were evaluated using a NanoDrop ND-1000 spectrophotometer (Thermo Fisher Scientific, Waltham, MA, USA).

For cDNA generation, RNA was diluted with nuclease-free water to standardize all samples. A master mix of 10× reverse-transcription buffer, 25× dNTP, 10× random primers, Multiscribe reverse transcriptase, RNase inhibitor, and RNase nuclease-free water from a high-capacity cDNA reverse-transcription kit with RNase inhibitor (all kit components from Applied Biosystems, Vilnius, Lithuania), was added at an equal volume to diluted RNA. Samples were placed in a PTC-200 Peltier Thermo Cycler (MJ Research, Waltham, MA, USA), which ran as follows: stage 1, 25 °C for 10 min; stage 2, 37 °C for 2 h; stage 3, 85 °C for 5 min; stage 4, hold at 4 °C.

Real-time PCR was carried out with predesigned TaqMan primers and FAM-MGB probes (Applied Biosystems, Pleasanton, CA, USA). All primers and probes were used in a 7500 Fast Real-time PCR system (Applied Biosystems). Genes were evaluated in triplicate with 2× TaqMan Gene Expression Master Mix (Applied Biosystems), 20× Custom TaqMan Gene Expression Assay Mix (Applied Biosystems), sample cDNA (50 ng/well for tissues, 100 ng/well for cells), and nuclease free water for a total of 10 μL per reaction well. A 20× pre-designed TaqMan Gene Expression Assay for RPS9 was used as endogenous control (Applied Biosystems). Thermal cycling conditions for the Fast 2-step PCR system were as follows: stage 1, 95 °C for 20 s; stage 2, 95 °C for 3 s; stage 3, 60 °C for 30 s, with 40 cycles of stages 2 and 3. Data were recorded and compiled using ExpressionSuite Software version 1.0.4 (Applied Biosystems) and analyzed using DataAssist Software version 3.01 (Applied Biosystems, Foster City, CA, USA). Data from in vitro cell samples, having no standard cell type for comparison, are expressed as 2^−ΔΔCT^. Efficiency (E) of PCR was calculated based on the slope of a standard curve of tissue samples, serially diluted. The slope of the standard curve was used to calculate E, using the equation E = (10^−1/slope^ − 1) × 100.

### 2.5. Reactive Oxygen Species

Quantification of ROS production was carried out using the commercially available OxiSelect fluorometric ROS assay (Cell Biolabs, San Diego, CA, USA). Briefly, BAEC were cultured in 96-well, black walled and clear bottom plates following the experimental design and the assay was carried out following manufacturer’s recommendations. Cells were preloaded with DCF-DA at 100 μM in serum-free media for 15 min before treatments were added. The assay utilizes a non-fluorescent probe, 2′,7′-dichlorodihydrofluorescein diacetate (DCFH-DA), which is cleaved by cellular deacetylases and is oxidized by ROS to yield the fluorescent and stable 2′,7′-Dichlorodihydrofluorescein (DCF) product. Temporal ROS production was determined by measuring fluorescence using a BioTek Synergy H1 plate reader (Agilent, Santa Clara, CA, USA) at 480 nm excitation and 530 nm emission wavelengths. Results were analyzed as the fold change in fluorescence signal over untreated controls.

### 2.6. Viability and Cytotoxicity

Evaluation of AEA effects on viability and cytotoxicity of BAEC was carried out using commercially available assay kits from Promega (Promega, Madison, WI, USA). Briefly, for multiplex evaluation of viability and cytotoxicity, BAEC were cultured for 18 h in 96-well white wall, flat bottom plates and treated following the experimental design. Viability was evaluated using the Promega CellTiter-Glo Assay, following the manufacturer’s recommendations. The assay is based on the principle that the amount of ATP generated is proportional to the number of viable cells. Luminescence was read on a BioTek Synergy H1 plate reader (Agilent, Santa Clara, CA, USA). A complimentary cytotoxicity assay from Promega was employed as a multiplex assay on the same plate and cells as the CellTiter-Glo assay.

### 2.7. Mitochondrial Function

#### 2.7.1. Cytochrome-C Release

Quantification of cytochrome-C released in response to AEA treatment in BAEC challenged with LPS was carried out using the Promega Cytochrome-C Assay (Promega, Madison, WI, USA). Briefly, BAEC were cultured in white 96-well plates following the experimental design and the assay was carried out following manufacturer’s recommendations. Luminescence was read on a BioTek Synergy H1 plate reader (Agilent, Santa Clara, CA, USA).

#### 2.7.2. Apoptosis via Caspase 3/7

Effect of AEA on apoptosis via caspase 3/7 of BAEC challenged with LPS was determined via a commercially available Promega Caspase-Glo 3/7 kit (Promega, Madison, WI, USA). Briefly, BAEC were plated in a white flat bottom 96-well plate and treated according to the experimental design, and the assay was carried out according to the manufacturer’s recommendations. Caspase activation of the substrate releases aminoluciferin and subsequent interaction of the free aminoluciferin with luciferase results in a luminescent signal proportional to caspase 3/7 activity. Luminescence was read on a BioTek Synergy H1 plate reader (Agilent, Santa Clara, CA, USA).

### 2.8. Targeted Lipidomic Analysis

#### 2.8.1. Solid Phase Extraction (SPE) of Cultured Endothelial Cells

Cell pellet samples collected from cell culture experiments, previously stored at −80 °C, were thawed while on ice and protected from light. Initially, cell pellet samples were hydrolyzed by incubating with a 3 M potassium hydroxide solution at 45 °C for 45 min, allowed to cool to room temperature before being acidified with hydrogen chloride to a pH range of 2–3. The cell pellet samples were centrifuged at 4816× *g* for 45 min at 4 °C and the supernatant was extracted in Phenomenex 8B S100 FCH extraction cartridges. After determining that cell pellet hydrolysis did not change detection of target metabolites, the hydrolysis step was not included in processing samples from subsequent experiments. After thawing, samples were centrifuged (4000× *g*, 30 min, 4 °C), and combined with HPLC-grade water and formic acid to achieve 20% methanol and maintain 0.1% formic acidification, respectively. Samples were extracted with SPE cartridges (Waters Oasis HLB 12 cc, 500 mg LP) preconditioned with methanol followed by HPLC-grade water, washed with 20% methanol solution and cartridges were dried for 15 min under full vacuum. A 7.5 mL 1:1 mixture of methanol and acetonitrile was used to elute extracted samples from the cartridges into glass tubes pre-coated with 10 μL of 20% glycerol in methanol followed by a 30 s drying under full vacuum. Samples were dried in a Savant SpeedVac (ThermoQuest, Holbrook, NY, USA) with an initial heating phase at 45 °C, residues suspended in 150 μL of a 4:1 (*v*:*v*) methanol and water mixture, and centrifuged (14,000× *g*, 5 min, 4 °C). The supernatant (120 μL) was transferred to an autosampler vial with a low volume insert and stored at −80 °C before LC–MS/MS analyses.

#### 2.8.2. LC/MS/MS Analysis

Details of LC/MS/MS analysis of isoprostanes are described in Mavangira et al., 2016 [15]. In short, the quantification of analytes was accomplished on a Waters Xevo-TQ-S tandem quadrupole mass spectrometer (Water Corp., Milford, MA, USA) using multiple reaction monitoring (MRM). Chromatography separation was performed with an Ascentis Express C18 HPLC column (10 cm × 2.1 mm; 2.7 μm particles, Sigma-Aldrich, St. Louis, MO, USA) held at 50 °C, and the autosampler was held at 10 °C. Mobile phase A was water containing 0.1% formic acid, and mobile phase B was acetonitrile. Flow rate was fixed at 0.3 mL/ min. Liquid chromatography separation took 15 min per sample. MRM parameters including cone voltage, collision voltage, precursor ion, production, and dwell time were optimized based on Waters QuanOptimize software (Waters Corp., Milford, MA, USA) by flow injection of pure standards for each individual compound.

The details of LC/MS/MS analysis for endocannabinoids are described in Williams et al., 2007 [16]. In short, the quantification of analytes was accomplished on a Q-Exactive Quadrupole-Orbitrap mass spectrometer (Thermo Fisher Scientific, Waltham, MA, USA) using multiple reaction monitoring (MRM). Chromatographic separation was performed with an Acquity BEH C18 UPLC column (10 cm × 2.1 mm; 1.7 μm particles, Waters Corp., Milford, MA, USA) held at 40 °C, and the autosampler was held at 10 °C. Mobile phase A was water containing 0.1% formic acid, and mobile phase B was acetonitrile. Flow rate was fixed at 0.3 mL/min. Liquid chromatography separation took 15 min per sample. MRM parameters including cone voltage, collision voltage, precursor ion, production, and dwell time were optimized based on Xcalibur Data Acquisition and Interpretation software (Thermo Fisher Scientific, Waltham, MA, USA) by flow injection of pure standard for each individual compound.

### 2.9. Endothelial Barrier Function Assessment

For assessment of endothelial barrier integrity, BMEC were plated on bovine collagen-coated 96-well plates with gold electrodes and cultured for 18 h. Electric currents passing through the monolayer were continuously measured by the Electric Cell-Substrate Impedance Sensing Z-theta system (ECIS Z-Θ, Applied Biophysics, Inc., Troy, NY, USA). Approximately 2 h prior to treatment addition, media were changed to serum free media. Trans endothelial resistance (TER) across the monolayer was monitored up to 32 h after LPS addition. TER was normalized to the time point immediately prior to LPS addition.

### 2.10. Statistical Analysis

For statistical analyses all data sets were tested for normality and variance, before *t*-test using proc “*t*-test” procedure and one- or two-way ANOVAs were performed using the proc mixed procedure, with or without repeated measures where appropriate using the SAS9.4 software (SAS Institute Inc., Cary, NC, USA). A Tukey adjustment for multiple comparisons was performed with a *p* ≤ 0.05 considered significant when all-pairwise comparisons were performed; otherwise, a Dunnett’s post-hoc correction was performed for comparison of different treatments to the untreated control. For statistical analyses, undetected measures of variables in a treatment class were presented as undetected and assigned a zero value for statistical analyses.

## 3. Results

### 3.1. LPS Alters ECS Related Gene Expression and AEA Biosynthesis in BAEC

**Figure 2:**
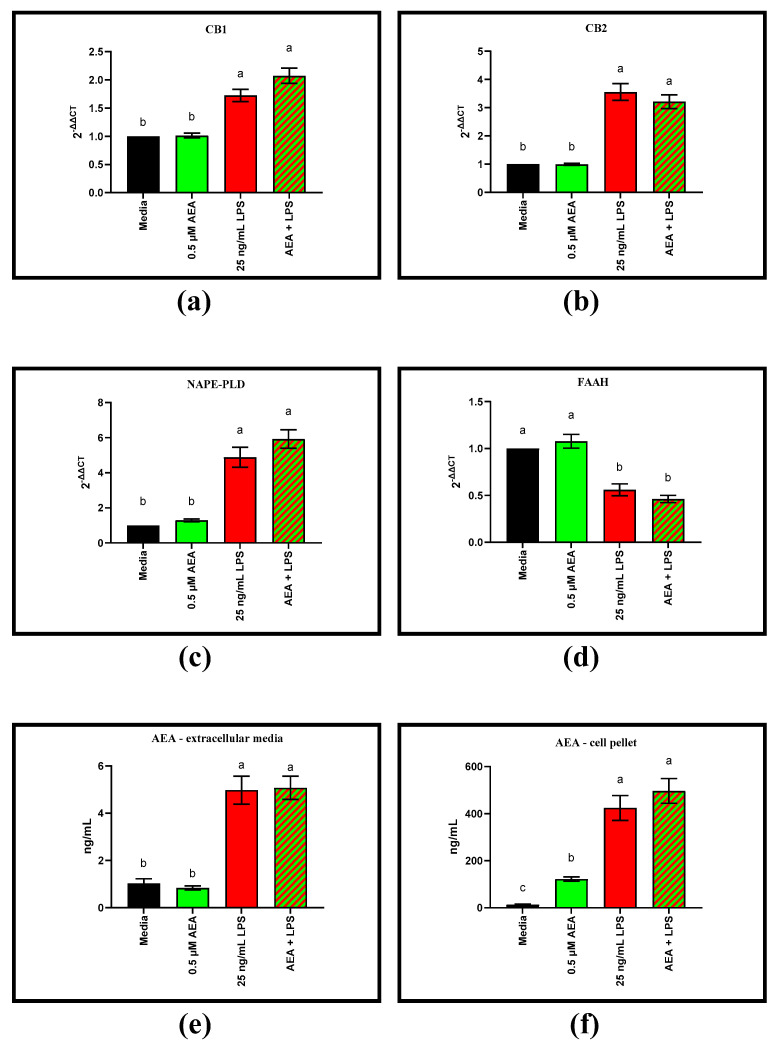
Gene expression via RT-qPCR of endocannabinoid receptors and arachidonoylethanolamide (AEA) associated genes in bovine aortic endothelial cells (BAEC) (N = 6). Extracellular and cellular AEA concentrations quantified via LC/MS. All results were recorded 8 h following lipopolysaccharide (LPS) challenge and/or 05. µM AEA: (**a**) gene expression of cannabinoid receptor-1 (CB1); (**b**) gene expression of CB2; (**c**) gene expression of N-acyl phosphatidylethanolamine phospholipase D (NAPE-PLD); (**d**) gene expression of fatty acid amide hydrolase (FAAH); (**e**) extracellular media concentration of AEA; (**f**) cell pellet concentration of AEA. Statistical analysis: one-way ANOVA, Tukey’s adjustment. a–c: Means with different superscripts are different (*p* < 0.05).

Analysis by RT-qPCR revealed that gene expression of CB1/2 and NAPE-PLD was elevated with LPS challenge (*p* = 0.0016, *p* = 0.0003, and *p* = 0.0010, respectively), whereas expression of FAAH decreased with LPS challenge compared to the media control (*p* = 0.0010). Addition of 0.5 µM AEA did not alter gene expression of CB1/2, NAPE-PLD, or FAAH, with and without 25 ng/mL LPS. (Figure 2a–d). Exposure of BAEC to LPS also increased intracellular and extracellular AEA concentrations (*p* < 0.0001 and *p* < 0.0001, respectively). Extra- and intracellular AEA concentrations did not change with addition of 0.5 µM AEA without LPS compared to the media only treatment. In the presence of 25 ng/mL LPS, 0.5 µM AEA does not alter extra- or intracellular AEA concentrations compared to the LPS control.

### 3.2. AEA Alters Endothelial Barrier Function

**Figure 3:**
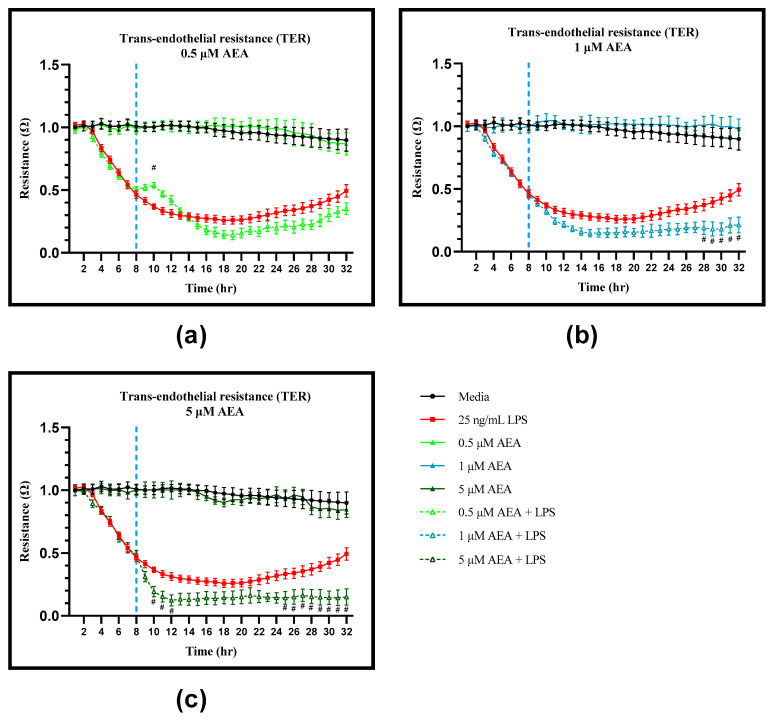
Analysis of trans-endothelial resistance (TER) utilizing electric cell-substrate impedance sensing (ECIS) of bovine aortic endothelial cells (BAEC) (N = 6) treated with (**a**) 0.5, (**b**) 1, and (**c**) 5 µM arachidonoylethanolamide (AEA), with and without 25 ng/mL lipopolysaccharide (LPS) pre-treatment. Statistical analysis: two-way ANOVA, Tukey’s adjustment. # *p* < 0.05 for AEA + LPS compared to LPS control.

Assessment of the trans-endothelial resistance (TER) via ECIS showed that without LPS pre-treatment, 0.5, 1, and 5 µM AEA did not alter barrier integrity compared to the media control (Figure 3). In BAEC pre-treated with LPS, addition of 0.5 µM of AEA increased TER at 10 h (*p* = 0.0457). Treatment concentrations of 1 and 5 µM AEA were detrimental to BAEC TER. Addition of 1 µM AEA resulted in decreased TER at 28, 29, 30, 31, and 32 h compared to the LPS control (*p* = 0.0410, *p* = 0.0107, *p* = 0.0024, *p* = 0.0028, and *p* = 0.0003, respectively), and with 5 µM AEA, TER was decreased at 10, 11, and 12 h (*p* = 0.0376, *p* = 0.0306, and *p* = 0.0298, respectively), and again at 25, 26, 27, 28, 29, 30, 31, and 32 h (*p* = 0.0282, *p* = 0.0302, *p* = 0.0268, *p* = 0.0083, *p* = 0.0020, *p* = 0.0003, *p* < 0.0001, and *p* < 0.0001, respectively) compared to the LPS control.

### 3.3. CB1 Mediates AEA Induced Changes in Endothelial Barrier Function

**Figure 4:**
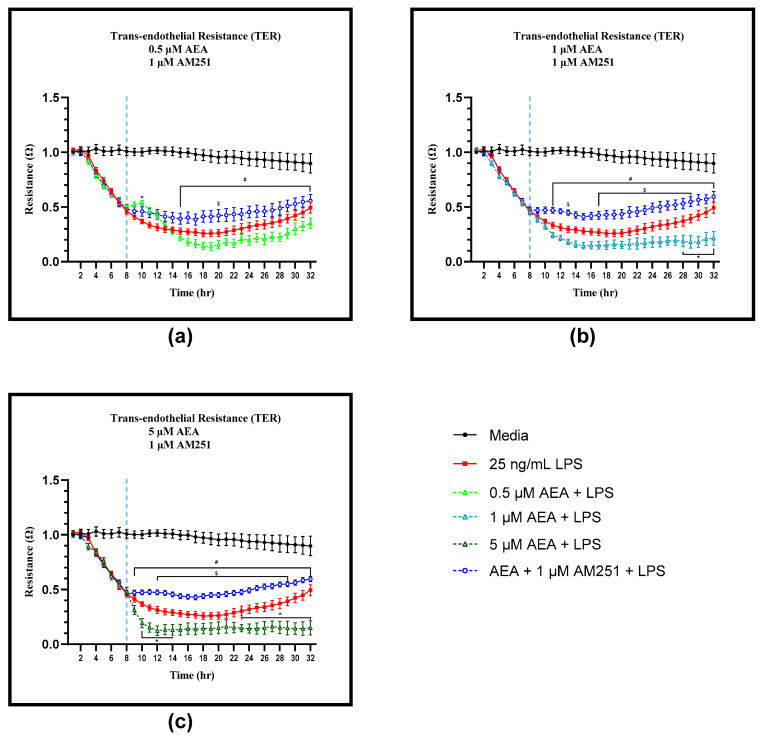
Analysis of trans-endothelial resistance (TER) utilizing electric cell-substrate impedance sensing (ECIS) of bovine aortic endothelial cells (BAEC) (N = 6) treated with 0.5, 1, and 5 µM arachidonoylethanolamide (AEA) and 1 µM cannabinoid receptor-1 (CB1) antagonist AM251 and 25 ng/mL lipopolysaccharide (LPS) pre-treatment. BAEC were treated with LPS at 0 h and AEA/AM251 at 8 h: (**a**) 0.5 µM AEA, 1 µM AM251, and 25 ng/mL LPS; (**b**) 1 µM AEA, 1 µM AM251, and 25 ng/mL LPS; (**c**) 5 µM AEA, 1 µM AM251, and 25 ng/mL LPS. Statistical analysis: two-way ANOVA, Tukey’s adjustment. * *p* < 0.05 significantly different to LPS control. # *p* < 0.05 AEA + AM251 + LPS treatment significantly different to corresponding AEA + LPS only treatment. $ *p* < 0.05 AEA + AM251 + LPS treatment significantly different to LPS control.

Treatment with 1 µM of the CB1 antagonist AM251 eliminated the 2 h TER increase associated with 0.5 µM AEA treatment in BAEC challenged with LPS (Figure 4a). Starting at 15 h, TER of BAEC pre-treated with LPS significantly increased with addition of 0.5 µM AEA + 1 µM AM251 compared to the 0.5 µM AEA only treatment. Treatment of LPS challenged BAEC with 0.5 µM AEA and 1 µM AM251 increased TER starting at 15 h compared to the AEA treatment alone and remained significantly greater throughout the 32 h timepoint (*p* < 0.05). Addition of 1 µM AM251 to 0.5 µM AEA increased barrier integrity over the LPS control at 20 h in BAEC pre-treated with LPS (*p* = 0.0460).

Similarly, LPS pre-treated BAEC had greater TER with the addition of 1 µM AM251 to 1 and 5 µM AEA, not only over the AEA only treatment, but TER of AEA and AM251 treatments was consistently greater than that of the LPS control (Figure 4b,c). Addition of 1 µM AM251 to 1 µM AEA increased TER compared to 1 µM AEA alone, starting at 11 h through to the 32 h timepoint (*p* < 0.05). Additionally, TER increased with 1 µM AEA and AM251 over the LPS control at 13 h (*p* = 0.0429) and again from 17 to 29 h (*p* < 0.05). Addition of 1 µM AM251 to 5 µM AEA increased TER of LPS pre-treated BAEC over 5 µM AEA only, starting at 9 h through to the 32 h time point (*p* < 0.05). Lastly, treatment with 5 µM AEA and 1 µM AM251 showed greater TER compared to the LPS control from 12 to 29 h (*p* < 0.05). By itself, 1 µM AM251 did not alter TER, with and without LPS (Graph S1).

**Figure 5:**
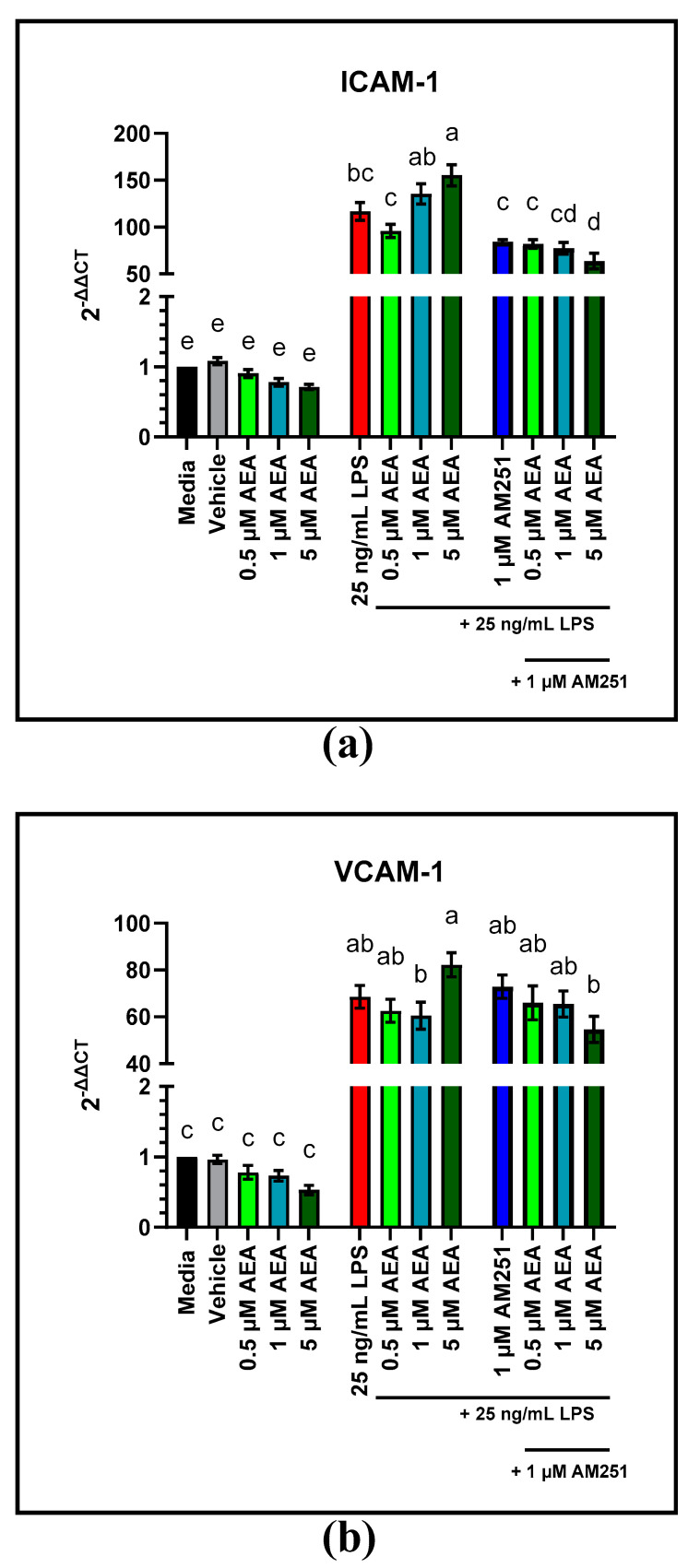
RT-qPCR analysis of gene expression of intracellular adhesion molecule-1 (ICAM-1) and vascular cell adhesion molecule-1 (VCAM-1) in response to 0.5, 1, and 5 µM arachidonoylethanolamide (AEA) and 1 µM AM251 treatment in bovine aortic endothelial cells (BAEC) (N = 6), with and without 25 ng/mL lipopolysaccharide (LPS) challenge: (**a**) expression of ICAM-1 following AEA and 1 µM AM251 treatment; (**b**) expression of VCAM-1 following AEA and 1 µM AM251 treatment. Statistical analysis: one-way ANOVA, Tukey’s adjustment. a–e: Means with different superscripts are different (*p* < 0.05).

RT-qPCR analysis of gene expression of ICAM-1 and VCAM-1 in BAEC not challenged with LPS was not altered by 0.5, 1, and 5 µM AEA treatment doses at 4 h, compared to the media control (Figure 5a,b). In BAEC pre-treated with LPS, only 5 µM AEA increased ICAM-1 gene expression over the LPS control (*p* = 0.0012), while VCAM-1 gene expression did not change with any AEA treatment concentration compared to the LPS control. However, in BAEC pre-treated with LPS gene expression of ICAM-1 was elevated following 4 h of 5 µM AEA treatment compared to 0.5 µM AEA (*p* < 0.0001). Similarly, expression of VCAM-1 in response to 4 h of 5 µM AEA treatment was greater than that of 0.5 and 1 µM AEA treatments (*p* = 0.0027 and *p* = 0.0007, respectively). Expression of ICAM-1 decreased with the addition of 1 µM of the CB1 antagonist AM251 to 0.5 µM AEA, compared to the 0.5 µM AEA only treatment (*p* = 0.0495). Expression of VCAM-1 was not altered by 0.5 µM AEA and 1 µM AM251. The remaining time points for ICAM-1 and VCAM-1 gene expression can be found in the Supplemental Data (Tables S7 and S8, respectively).

### 3.4. AEA Induced Endothelial Mitochondrial Dysfunction Is CB1 Mediated

**Figure 6:**
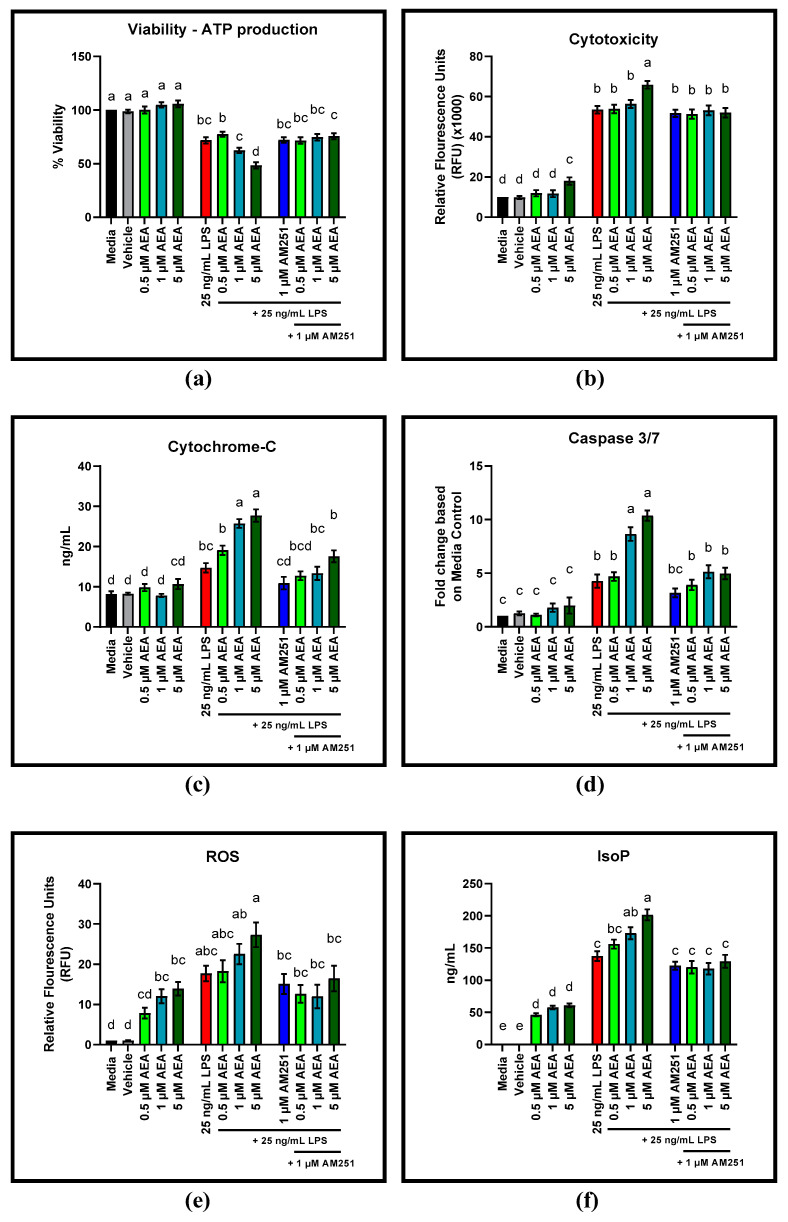
Viability via ATP release, cytotoxicity, cytochrome-c, and caspase 3/7 in bovine aortic endothelial cells (BAEC) (N = 6) treated with 0.5, 1, and 5 µM arachidonoylethanolamide (AEA) and 1 µM cannabinoid receptor-1 (CB1) antagonist AM251 for 4 h, in the absence and presence of 25 ng/mL lipopolysaccharide (LPS) challenge: (**a**) Viability via ATP production following AEA and 1 µM AM251 treatment; (**b**) Cytotoxicity following AEA and 1 µM AM251 treatment; (**c**) Cytochrome-C release following AEA and 1 µM AM251 treatment; (**d**) Caspase 3/7 activation following AEA and 1 µM AM251 treatment; (**e**) ROS production following AEA and 1 µM AM251 treatment; (**f**) IsoP production following AEA and 1 µM AM251 treatment. Statistical analysis: one-way ANOVA, Tukey’s adjustment. a–e: Means with different superscripts are different (*p* < 0.05).

AEA concentrations of 0.5 µM elevated ATP production in BAEC challenged with 25 ng/mL LPS compared to LPS alone (*p* = 0.0055) (Figure 6a). Addition of the CB1 antagonist AM251 did not alter ATP production in response to 0.5 µM AEA. Addition of 1 µM AEA did not alter ATP production, and addition of 1 µM AM251 to 1 µM AEA did not affect ATP release compared to the 1 µM AEA only treatment. However, 5 µM AEA decreased ATP release, compared to LPS alone (*p* = 0.0108) and addition of 1 µM AM251 increased ATP production compared to the 5 µM AEA only treatment (*p* = 0.0029).

Cytotoxicity was not affected by 0.5 and 1 µM AEA, but increased with 5 µM AEA compared to the LPS control (*p* = 0.0156) (Figure 6b). Addition of the CB1 antagonist AM251 reduced cytotoxicity of 5 µM AEA compared to AEA alone (*p* = 0.0045).

Cytochrome-C release from the mitochondria increased with all AEA doses used (0.5, 1, and 5 µM) compared to the LPS control (*p* = 0.0185, *p* < 0.0001, and *p* = 0.0001, respectively) (Figure 6c). The CB1 antagonist AM251 decreased cytochrome-C release at 1 µM concentration, compared to the LPS control (*p* = 0.0115) and addition of 1 µM AM251 to 0.5, 1, and 5 µM AEA reduced cytochrome-C release compared to the corresponding AEA only treatments (*p* = 0.0006, *p* = 0.0005, and *p* = 0.0045, respectively).

Activation of caspase 3/7 increased with the addition of all three concentrations of AEA (0.5, 1, and 5 µM) compared to the LPS control (*p* = 0.0004, *p* < 0.0001, and *p* < 0.0001, respectively) (Figure 6d). Addition of the CB1 antagonist AM251 reduced caspase 3/7 activation for all AEA treatment concentrations (0.5, 1, and 5 µM) compared to their respective AEA only treatments (*p* = 0.0006, *p* < 0.0001, and *p* = 0.0225, respectively).

Production of ROS is increased with LPS antagonism compared to the untreated control (*p* < 0.0001) (Figure 6e). Increase in ROS production with AEA treatment is not significant. However, addition of AM251 significantly reduced ROS production of LPS challenged BAEC treated with 5 µM AEA (*p* = 0.0480). Interestingly, addition of 1 and 5 µM AEA increased ROS production without LPS compared to the untreated control (*p* = 0.0404 and *p* = 0.0068, respectively. Production of IsoP without LPS challenge was increased with all AEA doses (0.5, 1, and 5 µM) (*p* = 0.006, *p* < 0.0001, and *p* < 0.0001, respectively) (Figure 6f). Addition of AM251 reduced IsoP production of LPS challenged BAEC treated with 1 and 5 µM AEA (*p* < 0.0001 and *p* < 0.0001, respectively), whereas IsoP production of 0.5 µM AEA was unchanged. The remaining time points for ATP-production, cytotoxicity, cytochrome-C release, caspase 3/7 activation, ROS, and IsoP production can be found in the Supplemental Data (Tables S1–S6, respectively).

### 3.5. AEA Induced ROS and IsoP Production in BAEC Is CB1 Medaited

**Figure 7:**
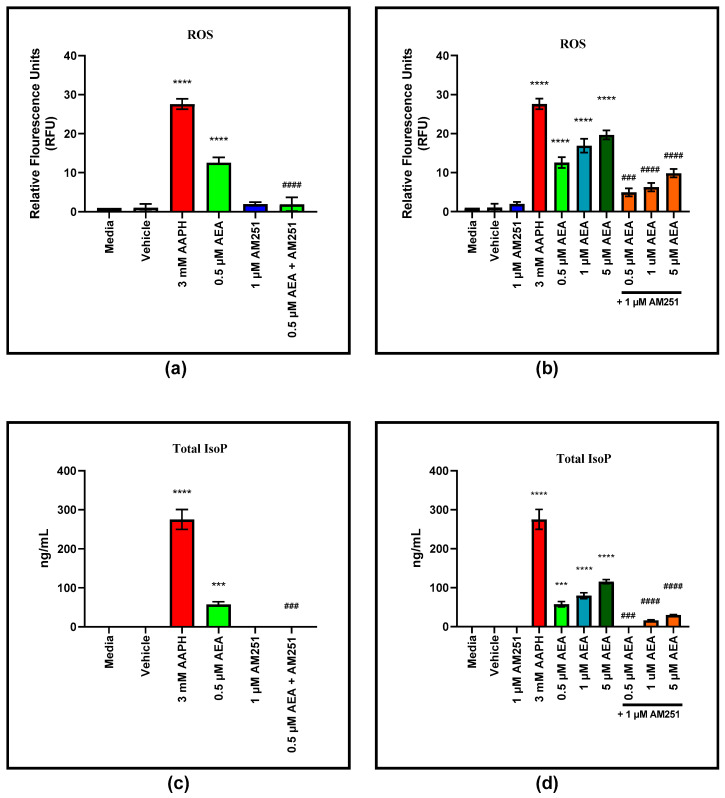
Reactive oxygen species (ROS) and isoprostane (IsoP) production of bovine aortic endothelial cells (BAEC) (N = 6) treated with arachidonoylethanolamide (AEA): (**a**) Involvement of cannabinoid receptor-1 and -2 (CB1/2) in ROS production of 0.5 µM AEA. CB1 antagonist: 1 µM AM251; (**b**) Dosage dependent ROS production of BAEC treated with AEA only, and combined with AM251; (**c**) Involvement of CB1/2 in total IsoP production of 0.5 µM AEA. CB1 antagonist: 1 µM AM251; (**d**) Dosage dependent total IsoP production of BAEC treated with AEA only and combined with AM251. Statistical analysis: one-way ANOVA, Tukey’s adjustment. * Significantly different to corresponding media control (*** *p* ≤ 0.001, **** *p* ≤ 0.0001); # Significantly different to AEA treatment of corresponding concentration (### *p* ≤ 0.001, #### *p* ≤ 0.0001).

Concentrations as low as 0.5 µM AEA induce ROS production in BAEC (*p* < 0.0001) (Figure 7a). Addition of 1 µM of the CB1 antagonist AM251 eliminated ROS production of 0.5 µM AEA (*p* < 0.0001). Production of ROS was dosage dependent and significantly elevated at 0.5 µM, 1 and 5 µM AEA compared to the media control (*p* < 0.0001, *p* < 0.0001, and *p* < 0.0001, respectively) (Figure 7b). Addition of 1 µM AM251 reduced ROS production of all three AEA doses compared to their respective AEA only treatments (*p* = 0.0002, *p* < 0.0001, and *p* < 0.0001, respectively). Total IsoP concentrations were also elevated over media control with 0.5 µM AEA addition (*p* = 0.0002) (Figure 7c). Addition of AM251 to 0.5 µM AEA eliminated IsoP production compared to the AEA only treatment (*p* = 0.0002). Total IsoP production is also dosage dependent, with 0.5 µM, 1 and 5 µM of AEA inducing IsoP production (*p* = 0.0002, *p* < 0.0001, and *p* < 0.0001, respectively) (Figure 7d). Addition of AM251 eliminates 0.5 µM AEA IsoP production (*p* = 0.0002), but only reduces total IsoP of 1 and 5 µM AEA (*p* < 0.0001 and *p* < 0.0001, respectively).

## 4. Discussion

In this study, we demonstrate that coliform mastitis endothelial barrier damage may include ECS activation as a mechanistic component. The activation of the endothelial endocannabinoid receptor CB1 mediates effects of AEA on endothelial barrier integrity and mitochondrial function. Addition of AEA altered gene expression of adhesion molecules, induced ROS production, increased cytochrome-C release, and activation of apoptotic pathways via caspase 3/7. However, treatment of endothelial cells with AEA without LPS challenge did increase ROS production and the likelihood of oxidative stress, but did not alter barrier function or activation of apoptotic pathways, indicating that inflammatory state and timing of AEA increase are crucial.

Basal expression of CB1 and 2 in this study indicates the involvement of the ECS in non-inflammatory functions of BAEC. Non-inflammatory models utilizing AEA in non-bovine species support the involvement of the ECS in a variety of processes in vascular endothelial cells, such as regulation of vascular tone [17] and angiogenesis [18]. Increased gene expression of the cannabinoid receptors following treatment with an antagonist such as LPS was demonstrated in many cell types, not just immune mediating cells. Expression patterns of ECS related genes observed in this study agree with endometrial expression of ECS related genes in Holstein dairy cows suffering from endometritis [10] and expression of hypothalamic ECS genes in Holstein dairy cows experiencing metabolic stress due to onset of lactation [19]. Therefore, elevated synthesis or release of AEA and receptor expression reported in this study, support involvement of the ECS in inflammatory regulation of BAEC.

Treatment of BAEC with up to 5 µM AEA without LPS challenge did not alter TER, whereas in the presence of LPS even nanomolar concentrations altered TER. Lack of change in gene expression of ICAM and VCAM-1 with 0.5 and 1 µM AEA addition stands in contrast to decreased expression of the adhesion genes in human blood-brain barrier treated with AEA in an ischemia/reperfusion model [20]. Simultaneous treatment with LPS and AEA may have different results to our study, not only due to cell type, but also timing. As LPS increases not only AEA concentrations but also CB1 expression and addition of AEA before or with LPS antagonism may result in degradation of AEA to arachidonic acid and subsequent metabolism to oxylipids, affecting expression of adhesion molecules. Additionally, supraphysiological concentrations of AEA may stimulate non-CB receptors, particularly prior to the upregulation of CB receptors following LPS antagonism. Furthermore, as demonstrated in this study, supraphysiological concentrations of 5 µM or greater of AEA may affect the survivability of endothelial cells, and the relative expression of adhesion molecules may no longer be the greatest contributor to changes in TER. Indeed, in human primary coronary artery endothelial cells, AEA induced a concentration- and time-dependent activation of MAPK, cell death, and ROS generation [21].

Even though ROS production was not significantly different with addition of AEA in this study, we did observe a numerical increase that was dose dependent, and addition of AM251 significantly reduced ROS production of 1 and 5 µM AEA, indicating that activation of the CB1 receptor by AEA does contribute to overall ROS production and possible mitochondrial dysfunction. Furthermore, addition of 1 and 5 µM AEA to LPS challenged BAEC increased IsoP production compared to the LPS control, which was ameliorated by the addition of AM251. Elevated IsoP production is an indicator for oxidative stress, or damage to healthy tissue by free radicals, such as ROS. Even though the effect of IsoP on bovine vascular endothelial cells remains elusive [22], increased ROS production is associated with endothelial dysfunction [23]. Elevated IsoP production associated with addition of 1 and 5 µM AEA may therefore be a key contributor to decreased TER observed at these concentrations. Increased release of cytochrome-c associated with 0.5, 1, and 5 µM AEA treatment of LPS challenged BAEC is indicative of mitochondrial dysfunction and initiation of apoptotic pathways. Elevated cytochrome-C release of LPS challenged BAEC treated with AEA was ameliorated by addition of the CB1 antagonist AM251, reducing subsequent activation of caspase 3/7 apoptotic pathways. However, 5 µM of AEA did not increase cytochrome-C release over 1 µM of AEA, but activation of caspase 3/7 did increase compared to the 1 µM AEA treatment, indicating activation of caspase 3/7 in a mitochondrial independent manner at micromolar concentrations of AEA. Interestingly, without LPS antagonism, even nano-molar concentrations of AEA are sufficient to induce ROS production and subsequent production of IsoP, indicating the presence of oxidative stress in BAEC. Presence of IsoP with only AEA addition, no LPS or other antagonist, highlights the sensitivity of mitochondrial metabolism to CB1 activation. Increased concentration of AEA following LPS exposure in BAEC and reduced expression of the AEA degradation enzyme FAAH may therefore be sufficient to increase cytochrome-c release with addition of only 0.5 µM AEA in this model of endotoxemia associated inflammation.

The compounding of increased AEA concentrations and CB1 expression may therefore lead to detrimental effects on barrier integrity and endothelial cell function following any additional AEA increase, for example by NSAIDs. Elevated AEA following NSAID treatment has been recorded in several models, in vivo and in vitro, and has been associated with analgesic and anti-inflammatory effects in several cell types. However, treatment of bovine mammary epithelial cells with the NSAID flunixin meglumine (FM), the only FDA approved NSAID for endotoxemia associated with acute coliform mastitis, significantly reduced TER for up to 10 h post treatment [24]. Elevated systemic AEA, decreased barrier integrity following NSAID treatment, and the data from this study showing that even nano-molar concentrations are sufficient to induce ROS production and alter TER, indicate that untimely increase in AEA concentrations during endotoxin challenge may be detrimental to endo- and perhaps even epithelial barrier integrity. However, further studies are needed to determine if elevated AEA associated with NSAID treatment is detrimental to the inflammatory response and endothelial function, and if timing is a factor.

Interestingly, 0.5 µM AEA treatment increased TER at 2 h but did not exhibit any changes in ICAM/VCAM expression or other parameters that were evaluated (see Supplemental Data). The increase in TER observed may be due to morphological changes, as the endogenous analog of AEA, N-arachidonoyl-L-serin modulates the cytoskeleton (actin) of the human cerebral microvascular endothelium [25]. Detrimental effects on endothelial cell function associated with 1 and 5 µM AEA treatment in this study may eliminate any potential TER increase. However, without morphological analysis, possibly through immunostaining, the cause of the 2 h increase in TER associated with 0.5 µM AEA cannot be speculated on here.

Future studies should focus on the involvement of CB2 and non-CB receptors, such as TRPV1. Non-published data from this study shows that antagonism of CB2 alters BAEC barrier function independently of AEA addition, and the data was therefore not included in this publication. However, AEA is capable of activating CB2 and modifying the effects of CB2 activation by other agonists via allosteric modulation, particularly at supraphysiological concentrations greater than 5 µM [26]. Furthermore, elevated AEA, by separate addition or associated with NSAID treatment, may provide additional AEA as a substrate for metabolism into active mediators, such as prostaglandin ethanolamides, or prostamides, and other ethanolamide derivates of cyclooxygenase, lipoxygenase, and cytochrome p450 pathways. Several AEA derived mediators have already been shown to modulate inflammation [27,28,29].

Overall, addition of AEA had detrimental effects on BAEC barrier integrity, elevated ROS production, leading to mitochondrial dysfunction highlighted by cytochrome-C release, and activation of apoptotic pathways, namely caspase 3/7. Reports on anti- and pro-inflammatory effects of AEA are numerous, and indication of pro-inflammatory effects in BAEC pre-treated with LPS are shown here. Nonetheless, further studies on timing and concentration dependent effects of AEA on BAEC function are needed, as activation of CB1 by AEA can reduce or increase ROS production, depending on cell type, timing of administration, and concentration.

There are limitations to our study design and functional assays. Utilization of the ECIS as a functional assay of endothelial barrier function allows for real-time determination of barrier integrity and advanced modeling thanks to multiple frequency electrical resistance and capacitance measurements. However, the embedded electrodes impede imaging of the cells after completion of the ECIS assay. Therefore, the mechanism causing increases in TER at 2 h post 0.5 µM AEA could not be determined with the data presented in this study. Future studies should determine the morphological changes associated with CB1 activation by AEA.

## 5. Conclusions

Increased AEA concentrations during coliform mastitis further deteriorates endothelial barrier integrity. Treatment of endothelial cells with AEA at physiological concentrations (0.5 µM) results in ROS production, increasing likelihood of oxidative stress and activation of apoptotic pathways. However, dynamic concentrations of cannabinoids and expression of related genes emphasizes the importance of timing of endocannabinoid treatment. Additional studies pertaining to the timing of concentration ranges of endocannabinoids and endocannabinoid related drugs are necessary to determine the true effect of AEA and other endocannabinoids on endothelial barrier function. Improved endothelial barrier function is a crucial facet of improving welfare and reducing mortality and cost associated with coliform mastitis.

## Data Availability

All data generated or analyzed during this study are included in this published article.

## Supplementary Materials

The following supporting information can be downloaded at: https://www.mdpi.com/article/10.3390/antiox11081461/s1, Table S1: Viability (ATP-production) of BAEC treated with AEA and AM251, with and without LPS (2, 8, 12, and 24 h); Table S2: Cytotoxicity of BAEC treated with AEA and AM251, with and without LPS (2, 8, 12, and 24 h); Table S3: Cytochrome-C release of BAEC treated with AEA and AM251, with and without LPS (2, 8, 12, and 24 h); Table S4: Caspase-3/7 activation of BAEC treated with AEA and AM251, with and without LPS (2, 8, 12, and 24 h); Table S5: ROS production of BAEC treated with AEA and AM251, with and without LPS (2, 8, 12, and 24 h); Table S6: IsoP production of BAEC treated with AEA and AM251, with and without LPS (2, 8, 12, and 24 h); Table S7: ICAM-1 gene expression of BAEC treated with AEA and AM251, with and without LPS (2, 8, 12, and 24 h); Table S8: VCAM-1 gene expression of BAEC treated with AEA and AM251, with and without LPS (2, 8, 12, and 24 h); Graph S1: Trans-endothelial resistance of BAEC treated with 1 µM AM251, with and without LPS.
