# Supplementary material for: Anandamide Alters Barrier Integrity of Bovine Vascular Endothelial Cells during Endotoxin Challenge
Source: Antioxidants (Basel). 2022 Jul 27;11(8):1461. doi: 10.3390/antiox11081461 (PMC9405077; doi:10.3390/antiox11081461)

Supplemental Materials:

Table S1: Viability (ATP-production) of BAEC treated with AEA and AM251, with and without LPS (2, 8, 12, and 24 hours).								
	2 hr		8 hr		12 hr		24 hr	
**Treatment**	Mean	SEM	Mean	SEM	Mean	SEM	Mean	SEM
Media	100.0000	0.0000	100.0000	0.0000	100.0000	0.0000	100.0000	0.0000
Vehicle	99.2731	1.7520	100.8842	1.5629	102.3911	1.4792	99.9362	1.7891
0.5 µM AEA	101.4192	2.9463	97.9030	3.1198	95.4717	3.8712	93.9856	4.0561
1 µM AEA	103.9571	2.8562	101.4820	2.5618	99.22815	4.7184	103.1779	5.2816
5 µM AEA	102.9129	3.0581	102.1354	3.9574	103.8136	4.0133	101.972	4.7945
25 ng/mL LPS	79.6392	2.0058	69.9947	2.5819	65.6811	3.1294	63.8732	4.6917
0.5 µM AEA + LPS	82.5801	3.5618	76.0937	3.6417	68.6592	3.5561	61.38756	3.3071
1 µM AEA + LPS	85.2789	3.8761	59.3472	4.0136	52.34523	2.5812	47.8163	4.0922
5 µM AEA + LPS	84.0966	2.9094	46.1829	3.7891	42.4897	3.5317	39.6493	4.1627
1 µM AM251 +LPS	75.0476	3.2372	73.9104	3.6801	74.22295	3.1093	75.0372	3.9761
0.5 µM AEA +1 µM AM251 + LPS	74.6107	4.5048	72.6192	4.0096	74.1389	3.6106	75.4436	4.7813
1 µM AEA + 1 µM AM251 + LPS	76.0154	3.6879	74.0816	3.6045	75.4617	4.0441	78.9467	5.1069
5 µM AEA + 1 µM AM251 + LPS	78.5881	3.0573	77.6545	2.6719	80.9516	3.5714	81.4812	4.7981

Table S2: Cytotoxicity of BAEC treated with AEA and AM251, with and without LPS (2, 8, 12, and 24 hours).								
	2 hr		8 hr		12 hr		24 hr	
**Treatment**	Mean	SEM	Mean	SEM	Mean	SEM	Mean	SEM
Media	10.0000	0.0000	10.0000	0.0000	10.0000	0.0000	10.0000	0.0000
Vehicle	11.0031	0.8462	9.5476	1.0573	10.3319	0.9441	9.0047	1.0515
0.5 µM AEA	9.5619	1.9561	12.3452	2.0351	13.4256	2.4871	14.4762	1.9021
1 µM AEA	12.4816	2.4295	13.6493	2.4617	14.5941	2.5515	14.7769	3.6411
5 µM AEA	14.6944	2.0563	18.8313	3.0786	19.8093	3.0175	19.5257	4.3897
25 ng/mL LPS	51.5621	2.1537	57.3429	3.1638	59.6782	3.3246	55.2346	4.1792
0.5 µM AEA + LPS	49.7819	2.3691	59.5436	4.1837	58.5423	4.5859	57.2987	5.0700
1 µM AEA + LPS	53.1437	2.5015	62.9476	4.2916	63.5821	4.5343	59.0034	4.4578
5 µM AEA + LPS	56.9813	3.3022	65.8992	3.8919	68.4712	5.4287	69.4009	4.3413
1 µM AM251 +LPS	46.8604	2.7493	53.5717	4.3817	50.8719	4.9347	51.6981	5.2108
0.5 µM AEA +1 µM AM251 + LPS	48.3457	3.6172	51.9661	5.0943	51.7726	4.7791	52.7894	4.9072
1 µM AEA + 1 µM AM251 + LPS	47.2019	3.5881	50.4615	4.1637	48.9487	5.1043	51.0019	5.9666
5 µM AEA + 1 µM AM251 + LPS	49.5173	4.1306	53.9908	5.0038	50.9094	4.3468	48.9587	4.1937

Table S3: Cytochrome-C release of BAEC treated with AEA and AM251, with and without LPS (2, 8, 12, and 24 hours).								
	2 hr		8 hr		12 hr		24 hr	
**Treatment**	Mean	SEM	Mean	SEM	Mean	SEM	Mean	SEM
Media	8.6476	0.0000	8.8154	1.3490	7.9961	0.4857	7.3492	1.4919
Vehicle	9.0192	0.5948	9.1491	0.7581	8.2043	1.5991	7.8495	2.8313
0.5 µM AEA	8.5096	1.2492	8.3243	1.9343	9.0305	2.1458	8.1391	2.5167
1 µM AEA	6.9291	0.9844	8.9181	0.9037	8.7183	1.4106	7.9466	2.0881
5 µM AEA	8.4048	2.7658	9.5933	2.1931	9.1048	3.1789	8.1097	3.1943
25 ng/mL LPS	13.3017	2.5681	16.5482	2.0384	19.9772	2.5691	17.5124	3.4407

0.5 µM AEA + LPS	17.5131	2.5953	19.3204	1.8875	19.3532	3.1044	18.5827	4.1656
1 µM AEA + LPS	18.2456	2.5617	20.1459	1.5903	20.4821	2.8173	19.5602	3.1039
5 µM AEA + LPS	21.7287	3.0583	26.3979	4.1738	21.0795	2.9104	19.8736	4.0571
1 µM AM251 +LPS	11.4802	2.9791	11.4286	3.1904	11.9139	3.5091	8.1761	3.8193
0.5 µM AEA +1 µM AM251 + LPS	11.0747	3.0065	12.8152	2.5678	11.7281	4.0082	10.1411	4.7615
1 µM AEA + 1 µM AM251 + LPS	14.5941	2.6344	15.1369	2.0538	14.7769	3.6179	13.4256	4.1203
5 µM AEA + 1 µM AM251 + LPS	17.6141	3.0471	16.4450	3.1294	16.0375	4.2076	15.1407	5.1997

Table S4: Caspase-3/7 activation of BAEC treated with AEA and AM251, with and without LPS (2, 8, 12, and 24 hours).

	**2 hr**		**8 hr**		**12 hr**		**24 hr**	
**Treatment**	Mean	SEM	Mean	SEM	Mean	SEM	Mean	SEM
Media	1.0000	0.0000	1.0000	0.0000	1.0000	0.0000	1.0000	0.0000
Vehicle	0.9021	0.2149	1.2019	0.3948	0.9923	0.3719	1.0318	0.3481
0.5 µM AEA	1.1036	0.3917	1.1949	0.4816	1.2018	0.6581	0.9836	0.3171
1 µM AEA	1.4005	0.5028	1.4257	0.5162	1.1409	0.7172	1.0381	0.5136
5 µM AEA	1.6542	0.9822	1.4981	0.4811	1.2647	0.7129	0.9934	0.7594
25 ng/mL LPS	4.3158	0.8536	4.4578	1.5417	4.7445	1.2481	5.0953	1.5361
0.5 µM AEA + LPS	4.2159	0.7553	5.2016	1.9938	5.1839	1.5618	4.7165	1.2491
1 µM AEA + LPS	5.0399	0.8059	7.2643	1.4172	7.1124	1.8739	6.8479	1.7282
5 µM AEA + LPS	6.4271	0.9387	9.1856	1.1099	8.7910	1.5251	8.8697	2.0124
1 µM AM251 +LPS	3.0567	0.9812	3.4066	0.8953	3.1838	0.9317	2.0840	0.8158
0.5 µM AEA +1 µM AM251 + LPS	2.9983	0.7484	3.3217	0.9182	2.9053	1.0381	2.7401	0.9416
1 µM AEA + 1 µM AM251 + LPS	4.1677	1.2943	5.8172	1.1381	4.1219	0.9471	2.9408	0.7244
5 µM AEA + 1 µM AM251 + LPS	3.9087	0.9541	5.1298	1.0461	4.6183	0.8492	3.0128	0.9881

Table S5: ROS production of BAEC treated with AEA and AM251, with and without LPS (2, 8, 12, and 24 hours).

	**2 hr**		**8 hr**		**12 hr**		**24 hr**	
**Treatment**	Mean	SEM	Mean	SEM	Mean	SEM	Mean	SEM
Media	1.0000	0.0000	1.0000	0.0000	1.0000	0.0000	1.0000	0.0000
Vehicle	0.9961	0.1034	0.9841	0.1385	1.0392	0.2859	0.9754	0.2498
0.5 µM AEA	7.5431	1.4701	10.5718	1.4026	13.4817	2.0577	8.9271	2.6783
1 µM AEA	11.7461	2.7566	14.1627	1.9715	14.9204	3.0121	15.0116	3.5886
5 µM AEA	12.5812	3.1045	17.4033	1.8561	17.5119	3.6055	16.0520	3.7221
25 ng/mL LPS	17.4092	2.7904	15.9004	2.2561	15.5938	3.7612	11.5702	3.6152
0.5 µM AEA + LPS	15.6206	4.8607	17.5923	4.6817	16.2458	4.4617	15.8162	4.5615
1 µM AEA + LPS	19.4721	3.6592	20.5371	4.0354	19.1232	3.8093	17.0589	3.4679
5 µM AEA + LPS	22.6903	4.0295	22.0662	5.9877	21.4144	4.1236	20.5980	4.0183
1 µM AM251 +LPS	15.8577	3.2886	13.8463	3.6163	13.0717	2.7709	15.2193	3.5592
0.5 µM AEA +1 µM AM251 + LPS	14.7079	2.2358	12.4917	4.4719	11.0375	3.9042	10.5131	3.6733
1 µM AEA + 1 µM AM251 + LPS	14.3129	3.1638	11.5812	4.3917	11.3656	3.4871	11.0654	4.0816
5 µM AEA + 1 µM AM251 + LPS	20.8312	4.3761	18.4311	3.0611	15.1369	4.5694	12.7287	3.5617

Table S6: IsoP production of BAEC treated with AEA and AM251, with and without LPS (2, 8, 12, and 24 hours).

	**2 hr**		**8 hr**		**12 hr**		**24 hr**	
**Treatment**	Mean	SEM	Mean	SEM	Mean	SEM	Mean	SEM
Media	0.0100	0.0000	0.0100	0.0000	0.0100	0.0000	0.0600	0.0021

Vehicle	0.0100	0.0000	0.0100	0.0000	0.0100	0.0000	0.1000	0.0274
0.5 µM AEA	5.9331	2.3172	55.3279	5.9022	32.5981	7.6617	14.0317	5.7726
1 µM AEA	6.0736	2.5489	79.5103	7.5340	64.5871	8.0214	67.5006	9.4129
5 µM AEA	19.0904	2.9567	115.6958	5.1320	97.0938	9.6052	82.9594	14.0298
25 ng/mL LPS	108.0581	7.4821	142.9510	8.0291	120.6091	11.5551	124.1405	16.4618
0.5 µM AEA + LPS	113.7992	5.9519	158.1267	9.9473	141.4352	10.5268	125.6018	20.3504
1 µM AEA + LPS	109.1833	6.4316	193.5319	11.5172	157.0238	13.8532	128.0155	19.8749
5 µM AEA + LPS	117.6012	6.0041	242.8511	14.5609	198.4019	15.1796	142.5972	27.3993
1 µM AM251 +LPS	84.6694	7.2356	129.5018	7.3461	106.1231	6.5061	102.3428	10.9238
0.5 µM AEA +1 µM AM251 + LPS	80.0281	6.7264	104.7691	13.6817	114.0923	11.8325	103.7906	14.4482
1 µM AEA + 1 µM AM251 + LPS	77.1957	7.3078	110.0823	11.5080	103.5012	9.1589	97.4311	17.9727
5 µM AEA + 1 µM AM251 + LPS	82.3281	11.0863	113.5428	9.0159	107.2481	10.4853	84.6914	14.4971

Table S7: ICAM-1 gene expression of BAEC treated with AEA and AM251, with and without LPS (2, 8, 12, and 24 hours).

	**2 hr**		**8 hr**		**12 hr**		**24 hr**	
**Treatment**	Mean	SEM	Mean	SEM	Mean	SEM	Mean	SEM
Media	1.0000	0.0000	1.0000	0.0000	1.0000	0.0000	1.0000	0.0000
Vehicle	1.1905	0.1392	0.9847	0.0941	1.1371	0.1812	1.2045	0.4918
0.5 µM AEA	1.2943	0.0848	1.1038	0.1043	1.0318	0.3148	2.4581	0.8918
1 µM AEA	0.8842	0.0910	0.9281	0.1197	0.9653	0.1263	1.9361	0.7863
5 µM AEA	0.7229	0.0746	0.9238	0.2381	0.9841	0.2598	2.0149	1.3902
25 ng/mL LPS	114.8774	13.5093	115.0459	11.5009	114.1448	10.5864	113.6220	13.5919
0.5 µM AEA + LPS	99.0206	11.0098	97.0924	8.5999	106.7432	11.0687	109.7372	12.5837
1 µM AEA + LPS	111.1432	14.1887	134.5883	14.4527	131.8584	13.0012	121.5713	15.4726
5 µM AEA + LPS	121.7838	12.6992	142.0624	12.5983	125.7202	14.1787	122.0364	11.0491
1 µM AM251 +LPS	89.5693	6.9091	84.2447	4.1662	85.4769	6.6528	88.8710	7.4193
0.5 µM AEA +1 µM AM251 + LPS	86.0039	8.1439	78.3191	7.5914	72.5018	5.1948	70.4174	8.1298
1 µM AEA + 1 µM AM251 + LPS	78.4559	9.0016	74.3734	6.2986	68.8882	9.5617	65.9318	11.8581
5 µM AEA + 1 µM AM251 + LPS	68.9391	12.3653	64.9913	8.1263	60.4918	10.4168	56.9371	13.0207

Table S8: VCAM-1 gene expression of BAEC treated with AEA and AM251, with and without LPS (2, 8, 12, and 24 hours).

	**2 hr**		**8 hr**		**12 hr**		**24 hr**	
**Treatment**	Mean	SEM	Mean	SEM	Mean	SEM	Mean	SEM
Media	1.0000	0.0000	1.0000	0.0000	1.0000	0.0000	1.0000	0.0000
Vehicle	1.0544	0.1354	0.9881	0.1239	1.0034	0.3918	0.9634	0.3027
0.5 µM AEA	0.8322	0.1450	0.8128	0.1048	1.1204	0.2907	0.9927	0.2205
1 µM AEA	0.9471	0.0832	0.8552	0.1218	1.0028	0.1946	1.1049	0.2487
5 µM AEA	0.8061	0.0906	0.7461	0.1049	0.9906	0.2381	1.2471	0.3812
25 ng/mL LPS	67.4719	5.6217	69.4236	5.0126	68.6522	6.9328	65.8127	7.5827
0.5 µM AEA + LPS	65.6108	5.0151	64.1948	5.1237	60.4953	5.8225	56.0018	7.1254
1 µM AEA + LPS	64.4009	5.9274	61.4017	4.9073	58.3918	5.2237	55.1376	6.3731
5 µM AEA + LPS	76.1482	4.9863	81.4781	5.1044	75.0217	5.6248	69.2636	6.6827
1 µM AM251 +LPS	71.4611	5.9823	68.0139	6.1782	65.1204	6.0260	62.9173	7.6213
0.5 µM AEA +1 µM AM251 + LPS	68.5901	8.5006	65.0471	8.9913	63.5955	9.4272	61.7496	11.4844
1 µM AEA + 1 µM AM251 + LPS	64.0105	6.4371	64.3627	7.1267	63.4172	8.8284	62.0564	9.5712

5 µM AEA + 1 µM AM251 + LPS	59.1298	5.9989	53.3453	6.0928	52.1687	8.8962	53.8947	9.0766

Graph S1: Trans-endothelial resistance of BAEC treated with 1µM AM251, with and without LPS.

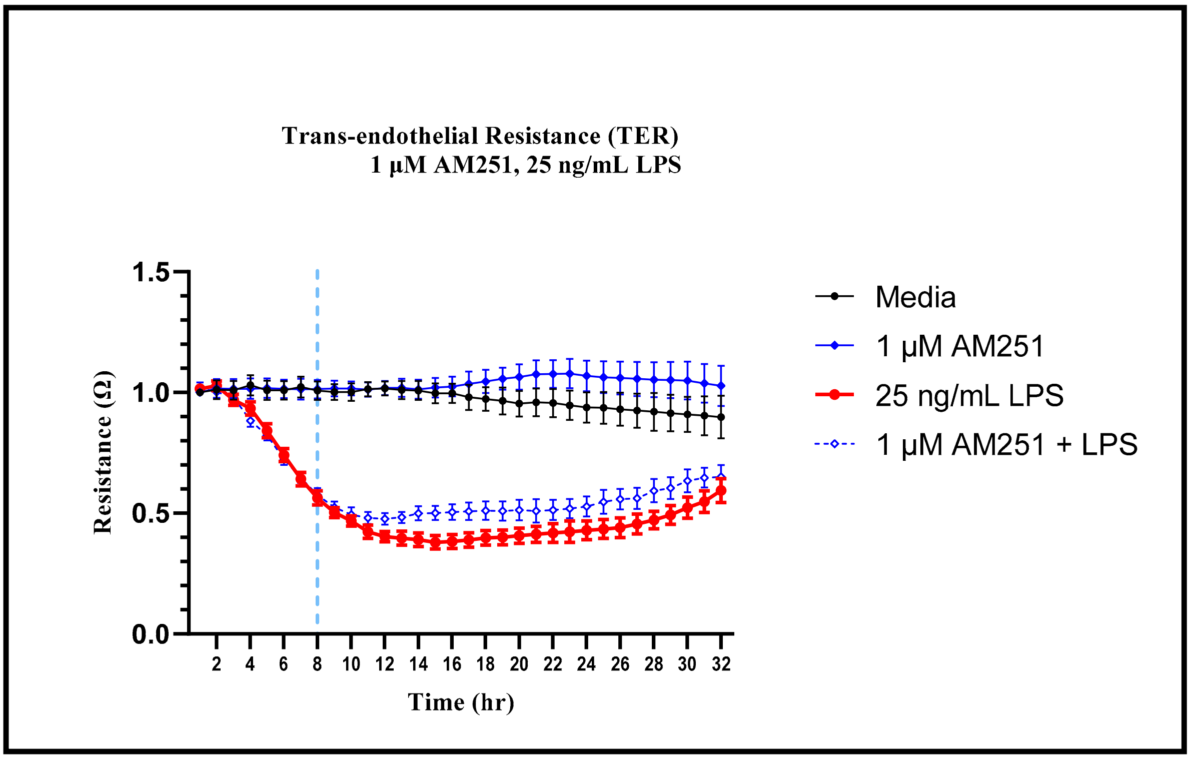

Supplement: Supplementary file 1 [file antioxidants-11-01461-s001.zip › antioxidants-1792878-supplementary.pdf]
